# Supplementary material for: Optimized Green Extraction of Polyphenols from Cassia javanica L. Petals for Their Application in Sunflower Oil: Anticancer and Antioxidant Properties
Source: Molecules. 2022 Jul 6;27(14):4329. doi: 10.3390/molecules27144329 (PMC9320193; doi:10.3390/molecules27144329)
Supplement: Supplementary file 1 [file molecules-27-04329-s001.zip › molecules-1778336-supplementary.pdf]

molecules

MDPI

Supplementary Data:

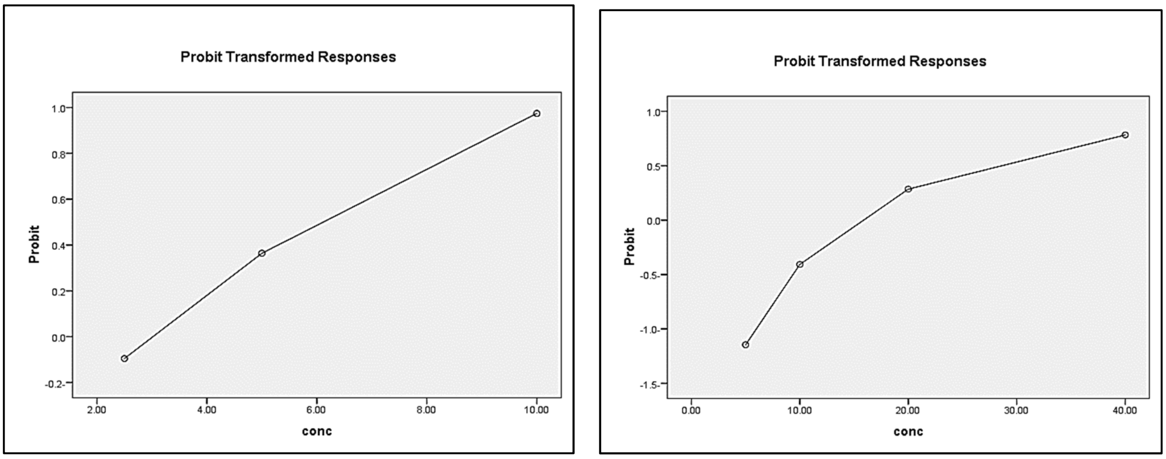

MCF7 PC3

Figure S1. The relationship between TPC concentration and the inhibition of cancer cells (breast, and prostate cancer)

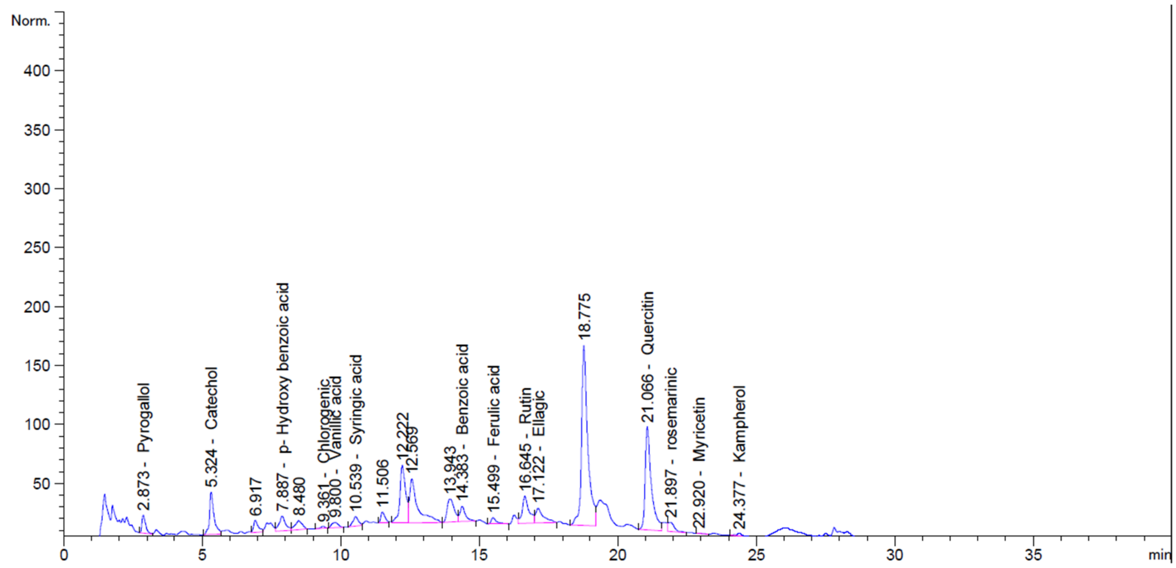

Figure S2. Solvent Extraction Chromatogram

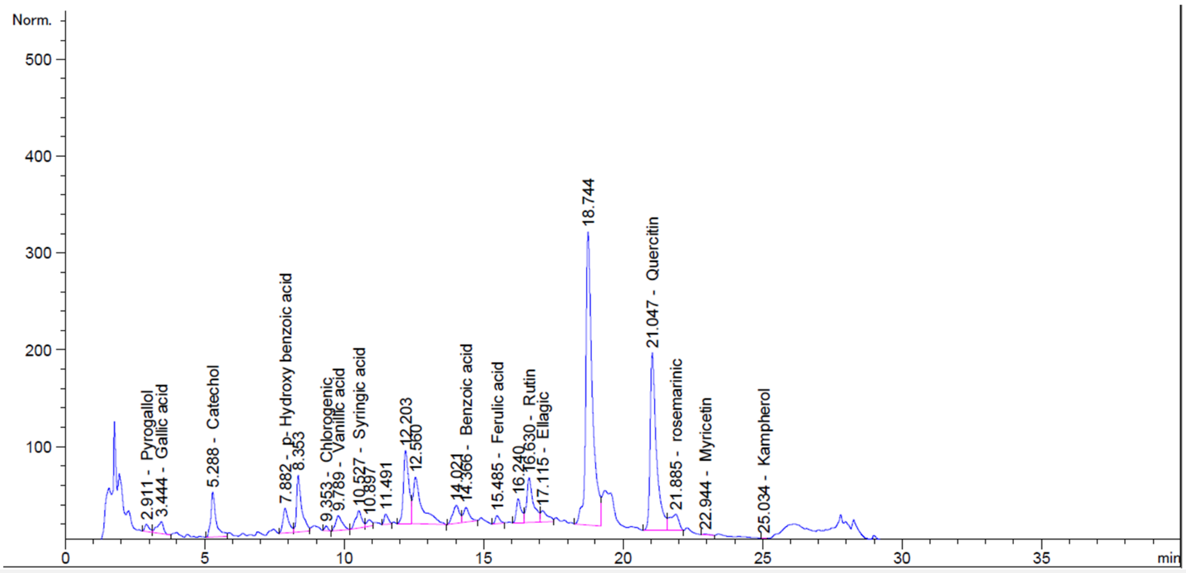

Figure S3. Ultrasound-Assisted Extraction Chromatogram

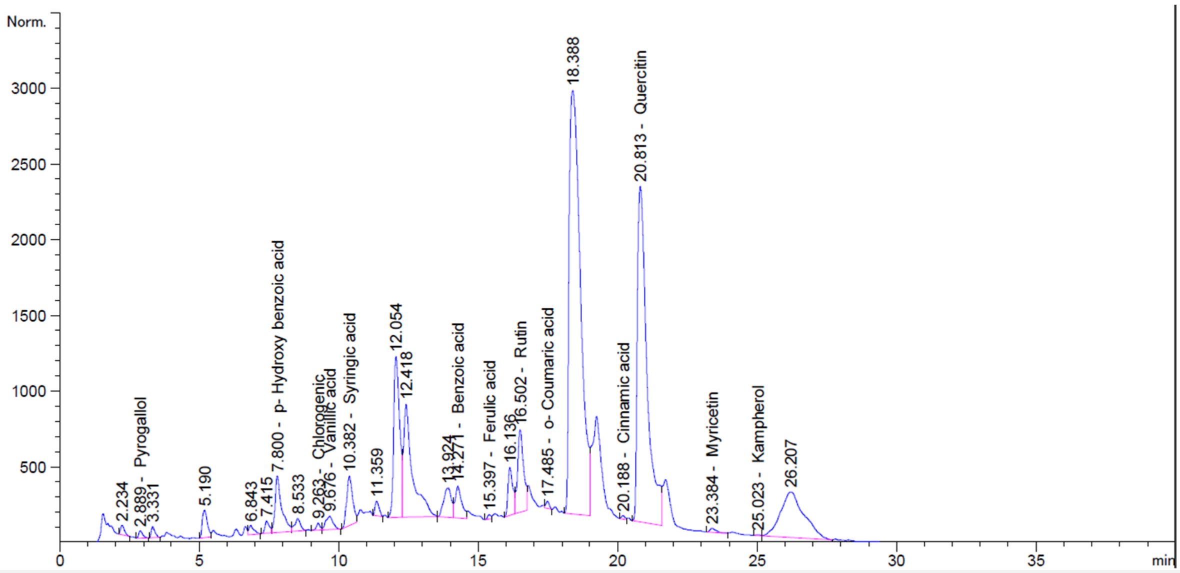

Figure S4. SCF-CO_2 Extraction Chromatogram
